# The silent protector: Nucleoporin93’s role in vascular health

**DOI:** 10.18632/aging.206097

**Published:** 2024-08-21

**Authors:** Julia Michalkiewicz, Tung D. Nguyen, Monica Y. Lee

**Affiliations:** 1 Department of Physiology and Biophysics, The University of Illinois at Chicago College of Medicine, Chicago, IL 60612, USA; 2 The Center for Cardiovascular Research, The University of Illinois at Chicago College of Medicine, Chicago, IL 60612, USA

**Keywords:** endothelial, vascular, aging, nucleoporin, inflammation

## Abstract

Nuclear envelope proteins have recently gained traction as novel regulators of endothelial and vascular function. Nuclear pore complexes (NPCs) stand as one of the largest protein complexes found at the nuclear envelope yet the role of component NPC proteins (i.e., nucleoporins) in vascular health remains unclear. In the issue of Aging Cell, Nguyen et al. (2024) identify Nucleoporin93, a major structural protein of the NPC, as an indispensable player in endothelial protection. This discovery raises the possibility that endothelial NPCs are susceptible to risk factors for consequent vascular disease.

Research in the last several decades has established endothelial cells (ECs) as a dynamic interface critical for vascular protection. Dysregulation of the endothelium promotes disease progression by triggering endothelial inflammation and vessel permeability, where subsequent changes in the underlying extracellular matrix allows for leukocyte entry and consequent cardiovascular disease (CVD) progression. Defined as a chronic state of systemic low-grade inflammation, human aging is a natural yet major risk factor for CVD onset. As such, endothelial dysfunction is the initial phenotype observed in the human aging process, contributing toward vessel inflammation, arterial stiffening, and impaired tissue-barrier function [1]. However, the molecular mechanisms by which EC aging promote vascular dysfunction and CVD remain elusive.

Endothelial health is largely dictated by the regulation of specific transcription factors that control the transcriptome and identity of ECs. Vessel inflammation and vascular aging are associated with increased Yes-associated protein (Yap) activity, a transcriptional co-regulator known to activate EC inflammation [2, 3]. In the established vasculature, Yap triggers inflammation by promoting the expression of senescence-associated and pro-inflammatory genes in ECs. Hence, nuclear accumulation of Yap is a key event for signal activation and downstream inflammation. Moreover, emerging studies demonstrate Yap activity as a major contributor to pathophysiological processes including atherosclerosis, a chronic inflammatory vascular disease [2, 3].

Transcriptional regulators, such as Yap, rely on nucleocytoplasmic shuttling, where subcellular localization determines signal outcome. The controlled exchange of molecules between the nucleus and cytoplasm occurs through nuclear pore complexes (NPCs), one of the largest components of the nuclear envelope [4]. The human NPC is a highly organized unit composed of ~30 different protein subunits known as nucleoporins to form a nucleocytoplasmic gateway. NPCs permit passive diffusion of small macromolecules (<40 kDa), whereas larger macromolecules depend on an active, carrier-based transport system that requires signal recognition by transport receptors known as karyopherins (e.g., exportins, importins) [4]. Previous groups have established nucleoporins as extremely long-lived proteins with limited turnover capacity in post-mitotic cells [5, 6]. As such, nucleoporin deterioration occurs as a major consequence of aging in long-lived cells such as neurons, where recent studies link NPC dysfunction to neurodegenerative diseases [7, 8]. Nucleoporin93 (Nup93) is necessary for the NPC structure and one of the most abundant nucleoporins in mammalian cells known to decline with age [5, 9]. In the issue of Aging Cell, Nguyen et al. provide striking evidence for age-associated nuclear pore component loss (i.e., Nucleoporin93 (Nup93)) in ECs, adding vascular cells to the growing list of cell types that exhibit nuclear permeability with age [5, 10]. Endothelial Nup93 protein levels were found to be significantly reduced in the vasculature of aged mice (17 months), paralleling observations of Nup93 loss when using *in vitro* models of senescence [10]. This observation illuminates the enigmatic role of NPC proteins (i.e., nucleoporins) to add to the emerging list of nuclear envelope proteins involved in EC behavior.

Mechanistically, Nguyen et al. report aberrant nuclear Yap accumulation as the driving factor for endothelial senescence in Nup93-deficient ECs. Confirming Yap as the major pathway triggered upon Nup93 loss, pharmacological inhibition of Yap activity was found to fully reverse the senescence-associated phenotypes initially seen in Nup93-deficient ECs. To examine how Nup93 loss affects NPC transport properties, the authors used a well-characterized reporter construct to visually measure nucleocytoplasmic transport of an artificially labelled fluorescent protein. Suboptimal levels of Nup93 led to a preferential nuclear accumulation of cargo protein to suggest impaired nucleocytoplasmic transport properties in senescent ECs. Intriguingly, re-expressing Nup93 protein levels was sufficient to reprogram senescent ECs toward a healthy endothelial phenotype, including a restoration of nucleocytoplasmic transport function [10]. These observations identify endothelial Nup93 as a key determinant of EC health, where aging targets endothelial Nup93 levels to impair NPC function as a novel mechanism of EC senescence and vascular aging.

The findings in Nguyen et al. also raise several interesting questions. For example, why are only certain nucleoporins susceptible to degradation? The selective loss of Nup93 implies that only certain endothelial nucleoporins acquire age-associated biochemical damage. Oxidative stress and/or NPC misassembly have been proposed as plausible mechanisms leading to the decline of select nucleoporins during aging [5, 11]. Future research will therefore require investigation into post-translational modifications (e.g., carboxylation, ubiquitination, phosphorylation) that may trigger protein degradation pathways (e.g., proteasomal, lysosomal) for a subset of endothelial nucleoporins. Similarly, how do suboptimal levels of Nup93 impact NPC stoichiometry, numbers and distribution? At the cellular level, age-associated NPC defects can include the displacement, mislocalization, and/or clustering of NPCs [8]. Applying high-resolution microscopy techniques to visualize NPCs at a single-cell level will certainly enhance our current understanding of how NPC properties influence EC health. Novel strategies to restore endothelial health continue to emerge as ways to improve cardiovascular longevity. An additional question: Does reestablishing NPC integrity via exogenous Nup93 delivery serve as a potential vascular senotherapeutic? While the *in vitro* studies indicated re-expression of Nup93 in senescent ECs was sufficient to restore both NPC function and EC health, it remains unclear whether endothelial Nup93 restoration improves vascular health. Targeted delivery of Nup93 in regions of endothelial dysfunction using precision nanomedicine techniques may effectively attenuate vessel inflammation and disease onset. Lastly, does endothelial dysfunction, and consequent vascular disease, initiate with NPC defects? The NPC plays a paramount role in the behavior of all eukaryotic cells by directing communication between the nucleus and cytoplasm. NPC surveillance mechanisms have been described in lower model systems (i.e., yeast) to suggest similar measures may exist in ECs, supporting the recent appreciation for endothelial resilience in safeguarding against physiological stressors to resist disease. Considering endothelial dysfunction occurs as the primary phenotype in human aging, the constant exposure to circulating CVD risk factors may result in an accumulation of NPC damage. This may eventually overwhelm resilience mechanisms, manifesting in various phenotypes of EC dysfunction to prime the vasculature for disease. Investigating how endothelial-targeted Nup93 loss impacts whole vessel physiology would begin to address the compelling idea that Nup93 deterioration occurs as an initiating event for CVD progression. This will, however, require the generation of novel mouse models to fully understand the physiological and translational implications of altering endothelial Nup93 levels. Regardless, the studies provided by Nguyen et al. serve as the first report implicating nucleoporins and impaired nucleocytoplasmic transport properties in endothelial dysfunction. These latest discoveries provide a fresh and innovative perspective of EC biology, highlighting NPCs as major regulators of EC health that may underlie mechanisms of vascular aging and disease progression.

## References

[1] Sun X, et al. Front Physiol. 2021; 12:693067. 10.3389/fphys.2021.693067 34220553 PMC8242592

[2] Wang KC, et al. Proc Natl Acad Sci U S A. 2016; 113:11525–30. 10.1073/pnas.1613121113 27671657 PMC5068257

[3] Wang L, et al. Nature. 2016; 540:579–82. 10.1038/nature20602 27926730

[4] Beck M, et al. Nat Rev Mol Cell Biol. 2017; 18:73–89. 10.1038/nrm.2016.147 27999437

[5] D'Angelo MA, et al. Cell. 2009; 136:284–95. 10.1016/j.cell.2008.11.037 19167330 PMC2805151

[6] Toyama BH, et al. Cell. 2013; 154:971–82. 10.1016/j.cell.2013.07.037 23993091 PMC3788602

[7] Mertens J, et al. Cell Stem Cell. 2015; 17:705–18. 10.1016/j.stem.2015.09.001 26456686 PMC5929130

[8] Cho UH, et al. Neuron. 2020; 106:899–11. 10.1016/j.neuron.2020.05.031 32553207 PMC9041311

[9] Ori A, et al. Mol Syst Biol. 2013; 9:648. 10.1038/msb.2013.4 23511206 PMC3619942

[10] Nguyen TD, et al. Aging Cell. 2024; 23:e14095. 10.1111/acel.14095 38348753 PMC11019141

[11] Rempel IL, et al. Elife. 2019; 8:e48186. 10.7554/eLife.48186 31157618 PMC6579512

